# Telemedicine consultations with physicians in Swedish primary care: a mixed methods study of users’ experiences and care patterns

**DOI:** 10.1080/02813432.2021.1913904

**Published:** 2021-05-11

**Authors:** Felicia Gabrielsson-Järhult, Sofia Kjellström, Kristina Areskoug Josefsson

**Affiliations:** a School of Health and Welfare, Jönköping Academy for Improvement of Health and Welfare, Jönköping University, Jönköping, Sweden; b Department of Behavioral Sciences, Oslo Metropolitan University, Oslo, Norway

**Keywords:** Primary health care, e-health, patient experiences, patient satisfaction, care patterns, mixed methods, telemedicine, Sweden

## Abstract

**Objective:**

The study aimed to explore users’ experiences and care patterns concerning telemedicine consultations with physicians in Swedish primary care from 2017 to 2019.

*Design and participants*: A mixed methods study involving 26 qualitative interviews with users of telemedicine consultations from a national sample, complemented by a quantitative registry study of data from 10,400 users in a Swedish region.

**Results:**

Users mainly described telemedicine consultations as a positive experience and perceived that the service met their current health care needs. Users also valued high accessibility, timesaving, and the contribution to ecological sustainability. Users felt competent about choosing when to use telemedicine consultations, most commonly for less severe health care concerns. This was confirmed by the quantitative results; only a few users had other care contacts within physical primary care before, or after, the telemedicine consultation, attended acute care or phoned 1177 Health Care Guidance.

**Conclusions:**

This study provides a rare account of users’ experiences of telemedicine consultations. Users expressed satisfaction with this up-to-date use of health care resources for them as individuals, the health care system, and the environment. Telemedicine consultations were perceived as efficient and safe according to users. In addition, the study shows a low degree of further physical contacts in primary care or in acute care related to the telemedicine consultations.

**Key points**
- Users have positive experiences of telemedicine consultations with physicians and experienced that the service had meet their actual needs for health care.
- Users were mainly satisfied with the service and highlighted the value of high accessibility.
- Users experienced that telemedicine consultants provided an alternative care service for mostly minor health problems, perceiving them to save time and resources for themselves, the health care system, and the environment.
- Most telemedicine consultations did not result in additional contacts with 1177 Health Care Guidance, physical visits to primary care, or acute health care.
- Telemedicine consultations with physicians were mainly used by persons aged 0–30 years and need to be further developed to suit other age groups.

## Introduction

In the past decade, e-health services and telemedicine have developed rapidly worldwide including in Swedish health care [1–6]. The concept of telemedicine (or telehealth) is described as an activity that allows long-distance patient and clinician contact, care, advice, intervention, monitoring, and remote admissions. Direct-to-consumer telehealth in which a patient has access to telemedicine consultations with a physician *via* video or text chat is an expanding service organized in both public primary care and private companies [1,5–7]. This emerging way of working is debated by the media, in research, politics and practice, as well as by organizations providing health care services [2,8–13] On the one hand, it seems to be a desirable solution to implement telemedicine services, following the Swedish government’s vision for e-health, stating that Sweden should be best in the world in the use of digitalization by 2025 [14]. Increased digitalization could support users and provide optimized support for health care professionals to ensure equal, accessible, and high-quality care and welfare [11]. On the other hand, it has been argued that telemedicine consultations will increase care consumption, lead to higher costs, and create inequalities [1,15].

### Telemedicine consultations in Swedish primary care

Swedish primary health care faces many challenges, such as waiting times for consultation, lack of patient centredness, and uneven distribution of services [14,16,17]. Reforms to create a more decentralized health care system [16,18,19], with increased choice of private and publicly managed primary care providers for patients [20,21], have not solved these problems. Telemedicine services can increase the potential choices for patients. Patients can seek care in primary health care centres other than those located in their region, waiting times and travel times are reduced and the individual has more freedom of choice regarding who to contact, when and how.

Telemedicine consultations with physicians have become popular among health service users [7,21]. In this study, telemedicine consultations are defined as a patient consulting a primary care physician *via* video or chat in a digital platform. Comparison of the use of telemedicine consultations in various international health care systems is problematic, because the cost to the individual for using the service compared with conventional primary care visits to a physician may influence the choice of service used, and this may also be influenced by the type of service that the telemedicine provider offers and health literacy in the population.

In a study based on Swedish data from 2016 to 2017, Ekman et al. has shown there are differences in the age and diagnoses of patients using telemedicine consultations compared with patients in conventional primary care. Younger people (>30 years) use the service often and older people (65+ years) rarely. Socioeconomic status and urban or rural residence are influencing factors [21]. Previous research varies on whether telemedicine consultations are safe, cost-efficient, decrease workload, and are of true value to users [15,21–26]. However, research investigating patient satisfaction, user characteristics and care-seeking patterns associated with telemedicine consultations in Swedish primary care is lacking, although there are some international studies [8,27–29].

### Users’ and professionals’ experiences of telemedicine consultations

When discussing the value of telemedicine consultations with physicians in primary care, the perspective of both the patients and the practitioners should be considered. Advantages for patients are increased flexibility, autonomy and minimizing use of time and resources; for physicians, benefits include reduced phone load, increased efficiency, time released for medical assessments, less-crowded waiting rooms and more precise communication in the triage process [27,30]. In a newly published study, Fernemark et al. [6] show that physicians perceive working with telemedicine consultations as flexible and with a high grade of autonomy, but it is not seen as something a physician can do full time if medical skills are to be maintained. However, other authors have claimed that physicians have a more negative attitude towards digitalization of health care than patients and health care system experts [10,31]. Users’ care patterns may also be influenced by the type of telemedicine consultations (text-based chat or video) and the physician’s competence and self-efficacy in providing care digitally; feelings of insecurity in this field may lead to additional consultations, and there are differences between video meetings and text-based consultations [22,26].

Digitalization of care has also been criticized and concerns have been raised regarding patient safety and whether telemedicine consultations lead to unnecessary overuse of care [1,11,28,32,33]. On the one hand, it is estimated in a Swedish study that telemedicine consultations can reduce the cost of primary care [26]. On the other hand, a Britsh study suggested that telemedicine primary care meetings have the potential to off-load ordinary primary care but may also lead to increased care costs [25]. Thus, there is mixed support for the value of telemedicine consultations. To sum up, telemedicine consultations are controversial, and there is a lack of Swedish studies from the patients’ perspective that can create a knowledge base for better decisions.

### Objectives

The study aimed to evaluate telemedicine consultations with physicians in Swedish primary care by exploring user experiences and care patterns.

## Study population and methods

The study has a mixed methods design, which offers useful tools for investigating complex processes and systems in health care [34]. The data were obtained from qualitative individual phone interviews with current users of telemedicine consultations from a national selection complemented by data collection from a quantitative registry with patients from the Jönköping Region. In this case, the quantitative data informed the data collection and the analysis of the qualitative part. Originally, this study was financed as a commission research project from The Swedish Association of Local Authorities and Regions (SKR) [7].

### Sample and data collection

#### Qualitative data

In early spring 2019, three primary care organizations provided information at their digital platform about the qualitative part of this study. These telemedicine services represented the public primary care organizations’ joint telemedicine consultation service in Region Jönköping and the two largest private, national primary care telemedicine consultation services. They invited users to get in contact with the first author (Felicia Gabrielsson-Järhult) if they were interested in sharing their experiences of having a telemedicine consultation with a physician. Users responding to the invitation to participate sent their name and e-mail address to Felicia Gabrielsson-Järhult who responded with written information about the study. If the user still wanted to participate, he or she e-mailed their phone number to Felicia Gabrielsson-Järhult to book an interview.

The main criterion for inclusion was having used a telemedicine consultation service with a physician between April and June 2019. The participants had to understand Swedish, and users were invited from both rural and urban areas from all parts of Sweden. A purposive sampling strategy was used to find informants who could contribute to the qualitative data collection. The aim was to find a variety of experiences among telemedicine service users: first-time users, several-time users, users of more than one type of telemedicine service, and different health care reasons for using the telemedicine consultation. Variety in gender, age, geographic region in Sweden was sought, and users from the three organizations had to be represented. The quantitative results show that parents of small children were a large group among users of telemedicine consultations. In the original sample description to the telemedicine service providers, this user group was not covered sufficiently; therefore, additional interviews with a selected sample covering this group were added.

Twenty-six interview persons (IPs) (16 women, 10 men, aged 18–73 years, some of them also parents of children in need of care) were included in the study (Table 1).

**Table 1.** Characteristics of the participants in the interviews.

Informant code number	Gender	Age (years)	No. of times a telemedicine app used	Contact person	
				Adult	Parent/child patient
1	Female	18	>1	x	
2	Female	63	1	x	
3	Female	54	1	x	
4	Male	73	>1	x	
5	Female	46	>1	x	x
6	Male	50	1	x	
7	Male	20	>1	x	
8	Female	55	>1	x	
9	Female	47	>1	x	x
10	Female	44	>1	x	
11	Male	18	>1	x	
12	Female	58	>1	x	
13	Female	26	>1	x	
14	Male	43	>1	x	
15	Male	25	1	x	
16	Female	43	>1	x	
17	Male	68	1	x	
18	Male	44	1	x	
19	Male	35	>1		x
20	Female	32	1	x	
21	Female	40	>1		x
22	Female	40	>1		x
23	Female	58	1	x	
24	Female	40	>1	x	x
25	Male	39	>1		x
26	Female	37	1		x

An interview guide with semi-structured questions was prepared by the research team and tested in three pilot telephone interviews (Table 2). All interviews took place at a time chosen by the informants after e-mail correspondence with Felicia Gabrielsson-Järhult. Informed consent was obtained and recorded. The interviews lasted 12–42 min and were audio-recorded and transcribed verbatim.

**Table 2.** The interview guide.

1.	You recently had contact with a doctor online. Do you want to tell us why you needed to consult a physician at that time: what concern or health problem was it that led you to make this contact?
2.	Was it the first time you sought care in this way?
3.	On whose initiative was it that you contacted a physician for a telemedicine consultation?
4.	How did you find information/become aware of this possibility?
5.	Was the telemedicine consultation for a need you had for yourself or for a child/relative?
6.	Before you had this doctor's appointment *via* the internet, did you have contact with another health care institution/activity, e.g. 1177 Health Care Guidance, the emergency department at the hospital?
7.	Can you describe how your physician’s visit went?
8.	Do you want to tell me how you experienced your doctor's visit?
9.	Are you satisfied with the visit as a whole?
10.	If yes… what was good?
11.	Was there anything you perceived did not work well?
12.	Did the doctor's visit result in a solution to your health problem/what you sought help with?
13.	What happened after your telemedicine physician's appointment? Have you had any contact/taken any action since, e.g. in primary care, sampling/laboratory, 1177 Health Care Guidance or the emergency room?
14.	Are you planning to have further telemedicine meetings with physicians in the future?
15.	Is there anything else about your experience of a telemedicine appointment that you would like to share that I may not have asked about or that you would like to tell me more about?
16.	If you were to compare your telemedicine consultation with a traditional physical appointment, what would you say are the pros and cons?
17.	Would you recommend another relative or friend to seek care in this way?

#### Quantitative data

The quantitative data were derived from Region Jönköping, which has a population of 362,000. The sample population was estimated to be the same as the national average concerning gender and there was a small difference with regards to age; Jönköping Region has 5.7% of citizens aged 80+ years compared with 5.1% in Sweden. The Jönköping Regional Registry included data from users of telemedicine consultations from three primary health care organizations, the same organizations where the qualitative data was collected. The sample consisted of all telemedicine consultations from September 2017 to January 2019, covering 17,301 telemedicine consultations, of which 15,211 were with physicians only, for 10,400 individuals.

The quantitative data collection was performed together with workers at the regional office, and statistical consultant decisions were made concerning relevant time intervals for the individual’s care process. The researchers decided to set the following boundaries for the care process: contact with 1177 Health Care Guidance within 24 h before the telemedicine consultation, regular physical visit in primary care 7 days before or after the and a visit to acute care within 24 h after the telemedicine consultation. The deidentified data were extracted by the regions’ controllers and analysed by the researchers.

#### Data analysis

The qualitative data were analysed in accordance with the six steps of thematic analysis inspired by Braun and Clarke [35]: (1) thorough reading of each interview to become familiar with the data to get a sense of the whole; (2) generating initial codes in a systematic fashion across the complete data set aiming to find interesting quotes in relation to each code; (3) merge codes into themes that appeared in the analysis; (4) compiling the results and reviewing themes; (5) defining and naming themes; (6) final analysis and write up of the results. Each step was performed by Felicia Gabrielsson-Järhult and Prof. Kristina Areskoug-Josefsson and the analysis first generated four themes. Thereafter, Prof. Sofia Kjellström reviewed the data again together with Felicia Gabrielsson-Järhult and the analysis was developed further, ending with three themes. Finally, the authors compared the main findings with the quantitative data and had an analytical discussion to achieve consensus.

The quantitative analysis was performed using IBM SPSS Statistics version 24.0 (IBM Corp, Armonk, NY).

#### Ethics

The study received ethical approval from the Swedish Ethical Review Authority, reference number 2019-00291.

## Results

The qualitative analysis revealed three themes describing users’ experiences of telemedicine consultations with physicians: (1) meeting health care needs through accessibility, (2) users’ competent choices, and (3) users’ satisfaction with telemedicine consultations.

### Theme 1: meeting health care needs through accessibility

The users considered quick access to health care, not having to take time off work and reduced travel time to be the most positive aspects of telemedicine consultations. The possibility to book a consultation at a suitable time for the individual was especially valued. An older informant expressed:

I Googled, as they so popularly call it, and then I came across (name of telemedicine consultation provider). I thought that I could try and see how it worked. I downloaded the app on my computer; it was really easy. I was impressed by how fast it was, and that this procedure was so effective. IP4, male, 73 years

Informants mentioned needs in relation to personal finances and time, if they had to take time off from work to go to a consultation at a primary care centre. For people who have to commute long distances, the time inefficiency was leading to delays in seeking traditional face-to-face primary care. The decrease in travelling was mentioned as a positive aspect of telemedicine consultations not only from an individual perspective but also as a way to make a positive contribution to societal need for ecological sustainability.

Physical visits to primary care were considered old-fashioned and an ineffective use of resources. The booking and contact systems used in conventional primary care were seen by the informants as obstructive and strengthened the view that it is difficult to get an appointment with a physician.

Digitally, I can contact at any time when I feel like it. I really need to check on this and it is not always possible to be away from work or call and then wait to get a call back and then just get the answer that there is no doctor available today. You have to call again tomorrow when we open in the morning. And then maybe you miss calling in the morning because you have a meeting or you are running late or whatever, but when it comes to telemedicine consultations, I can just log in and pose my questions. I am single with three children, so there is a lot to be considered, and this works for me. IP9, female, 47 years

Some informants were travelling when they experienced acute care needs for themselves or their children; for example, they forgot their asthma or allergy medications. Telemedicine consultations have also been used when informants have been abroad, and they wanted to get a second opinion on whether prescriptions by a local physician were correct and in line with Swedish health care practice.

This accessibility enables parents to seek care for concerns about a child’s health that might not have been done otherwise until it had gotten worse. Parents appreciated that they could stay at home with their child rather than go to a care facility, especially if the child had an infection or they had to find a carer to watch a sibling. Teenage parents mentioned the problem of getting a teenager to go to a primary care centre; it is easier to get the teenager to seek care with a telemedicine consultation, possibly send photos of the problem and then book a consultation without delay.

A few informants also acknowledged that high accessibility may create a higher demand for care, and that some people might use it for minor or less urgent matters. Most users described they had a clear picture of what to expect from a telemedicine consultation and when it could be right to use it.

Of course, you can’t use telemedicine consultations for everything, but for the things that I have used it for, it has worked very well. Yes, it [telemedicine consultation] works better for me than conventional primary care visits. IP9, female 47 years

Some informants reflected on economic aspects, and there could be a risk for profitable companies to take advantage of high accessibility to charm new users.

It is 2 minutes, and then you have prescription. […] it feels quite money driven if I am to be honest. So there is a big financial gain in having a fast service because then you can feed off as many patients as possible. IP14, male 43 years

### Theme 2: users’ competent choices

The informants described that they felt competent to self-assess their health problem to ensure that telemedicine consultations were suitable. In simpler cases, the informants mostly had knowledge of what kind of support they needed from the physician. When the informants were unsure if a telemedicine consultation was the correct choice, the physician assisted them in this choice, which led to a sense of security.

I have chosen the type of errands that I think can work and are suitable for this type of service. Of course, I have had conventional appointments with physicians’ as well during this time for things that I don’t think are suitable for telemedicine consultations; but for simpler problems I think this is a great service. IP5, female, 46 years

The users reasoned that telemedicine consultations were a way to off-load mostly minor health problems and administrative tasks (e.g. renewal of prescriptions and medical certificates) from primary care.

I am thinking on a political level if this is something that is seen as the future and as a way for the health care system to provide better accessibility but also relieve the health centre of the patients who do not have to go there. ……. Yes, but it is interesting, because as long as primary care can be off-loaded in a positive way, you can improve care as well. IP10, female 44 years

When the physician decided that the health problem could not be assessed or helped through a telemedicine consultation, the patient was recommended or referred to traditional primary care or acute care facilities. In those cases, the informants considered that they had been given enough information to understand the physician’s decision, and most informants experienced this as adding feelings of safe, relevant and trustworthy care.

I had called because I was unsure what to do when my stomach pain was so bad, and I didn’t know why. Then the physician told me to go to the primary care centre. IP1, female, 18 years

However, there were also a few cases where the informants expressed different opinions, e.g. critique against extra revisits and not being prescribed certain medications for longer time periods. They also experienced deficits for patients with comorbidity. One man explained that he did not feel that they had really taken the time to listen, even though he had tried in his written communications triad.

I opened up a discussion about my stress and the life I live. There was an opening for being able to talk about it, but I experienced nil response. It was not even noticed; she prescribed the same medicine, and I never met with her again. I14, male, 43 years

The telemedicine consultations were seen by users as complementary to other services, such as visits to primary care, 1177 Health Care Guidance and acute care. All informants had knowledge and previous positive experiences of 1177 Health Care Guidance with nurses and the 1177 web information, but had concerns about accessibility, e.g. long waiting time on the phone. Only a few informants had contacted 1177 Health Care Guidance before booking the telemedicine consultation. The informants’ motives for their choice were that they had assessed the health problem as something that a nurse could not solve; e.g. decisions on antibiotics, prescriptions, and medical certificates that required a physician.

### Theme 3: users’ satisfaction with telemedicine consultations

An overall finding is that the informants were very satisfied with the telemedicine consultations. Many of the informants had talked to family members and friends about their positive experiences of how easy and professional their contacts with telemedicine consultations were and would recommend the service to others. Few mentioned that close relatives had used the service, and particularly not older family members.

The telemedicine consultations were either in the form of a chat or a video meeting. The impression was that the users had experienced a professional and respectful encounter regardless of form.

I felt that they had prepared before the consultation and prescribed something that they [the physicians] felt safe with for the health problem. IP27, female, 37 years

There were also advantages and disadvantages with various types of telemedicine consultations experienced by the users. One informant described it as nice to have the consultation *via* chat, because it was more anonymous, but other expressed disappointment about not meeting in person. An advantage of the chat was that the conversation was well documented, and easy to refer back to and see what had been said. Other informants described video consultations as trustworthy and creating feelings of safety. Seeing the physician online was described as increasing the feeling of a real meeting, equal to an appointment at a primary care centre.

Different experiences were revealed in relation to telemedicine consultations compared with conventional primary care appointments.

The negative side of using telemedicine consultations is that they do not know who I am, and this makes the meeting less personal. There is no real contact and no follow-up, so it may be better to use conventional primary care when there is a need for that. IP5, female 46 years

When telemedicine consultations were used for administrative tasks, such as renewal of prescriptions, or simpler health problems such as skin rashes, continuity was not seen as important, especially when the health problem was solved by a single telemedicine consultation. One informant who had changed location had been placed on a 3-month waiting list in the new city. When she needed prescribed medication and care urgently, the digital specialized physician worked in conjunction with her previous specialized care, assisted with prescriptions and gave the informant a referral that lessened the waiting time to specialized care.

#### Care patterns evolved from the quantitative data

The registry data showed that only 2.9% (10,400 individuals) of the inhabitants in Jönköping County had used telemedicine consultations with primary care physicians between September 2017 and January 2019. The sample included 15,211 telemedicine consultations with physicians. Most users, 90%, had only used telemedicine consultations with a physician, 76% only one telemedicine consultation (14% more than one). Ten individuals (0.09%) had >20 telemedicine consultations.

Few users had any care contact other than the telemedicine consultation; 4.0% had had telephone contact with 1177 Health Care Guidance, 1.0% had a following visit at an acute clinic, and 3.5% had a physical visit to a primary care centre (Table 3).

**Table 3.** Care patterns among users of telemedicine consultations with a physician (TCP) (*n* = 10,400).

Type of care contact/consultation	%	Time when data applied
1177 Health Care Guidance^a^	1.5	Within 24 h before TCP
Physical visit to primary care	4.0	Within 7 days before TCP
Physical visit to primary care	3.6	Within 7 days after TCP
Visits to acute care	1.0	Within 24 h after TCP
Only one telemedicine consultation	75.8	
Only telemedicine consultation, but more than one appointment	14.1	

^a^Each user can have more than one consultation with conventional physical visits at primary care, acute care, or 1177 Health Care Guidance.

The users were mainly aged 0–30 years, 68% including parents using the service for their children, and 2.5% were elderly (>65 years). The gender distribution was 60% women and 40% men. The most common diagnoses were acute upper respiratory tract infection, rashes, and coughs. Approximately 1400 different diagnoses were registered, and the 10 most common covered 20% of the telemedicine consultations.

## Discussion

The main result is that users experienced telemedicine consultations as an alternative health care service mostly for less severe health problems, and that they felt competent in choosing when these consultations were needed. These mostly positive experiences are complemented by care patterns that seem to support that the health needs of users are satisfactorily taken care of with telemedicine consultations. The use of other health care services, such as 1177 Health Care Guidance or physical primary care visits, was limited, at least for the same health concerns as dealt with in the telemedicine consultations. The debate in Sweden [12,15,33] and in the United States [1] concerning telemedicine consultations has focused on whether this health care utilization represents ‘new’ health care utilization. This relatively new service is regarded by some people as an excess utilization, or even unnecessary usage of health care resources. In this study, we aimed to describe care patterns and users’ experiences, and, therefore, we have not undertaken any analysis or estimation of whether the usage is on an acceptable level due to health economics or relevant with regard to the medical aspects.

### Users’ experiences of telemedicine consultations

One of the main reasons for using telemedicine consultations was that the service matched the users’ perceived needs. High accessibility is an influencing factor for choosing telemedicine consultations according to the users in our study and in previous research [24,28]. However, high accessibility does not mean that patients’ needs can always be met. Patients with comorbidities or complex diseases that require specialist care can normally not be assessed in a telemedicine consultation with a physician; informants seemed to be aware of this. The users experienced telemedicine consultations as safe and trustworthy, but other studies have raised the issue of potential risks of lack of quality of e-health services, which may lessen patient safety [2,36].

Some of the users experienced increased continuity and having the same physician throughout telemedicine consultations, but others did not experience continuity. However, the importance of continuity varied among the users, depending on the complexity of their health problem. Women used telemedicine consultations more often than men in our study, which is in line with previous results from Sweden [21,23]. Men are in general less likely to seek health care than women [32,37].

Parents, the largest group of consumers, particularly seemed to appreciate the service. The parents described that it may be easier for their children to accept the physician in a telemedicine consultation than in a physical consultation, given children’s familiarity with online tools. Parents with small children value telemedicine consultations highly, because they consume care regularly and, thus, want to decrease their need to stay away from work, find childcare for siblings, and be able to make their family life work as smoothly as possible. In a study of the health care behaviour of parents of young children, it is stressed that even though they use the internet as a health information resource, they still need to consult a physician for reassurance on how to care for their child [38].

The introduction of telemedicine consultations in primary care has been a controversial issue due to varying political opinions concerning the use of telemedicine [12]. When this study was conducted, there still was a negative attitude in Swedish society about telemedi cine services. The Covid-19 pandemic has rapidly changed the conditions and context for telemedicine consultations and many primary care centres have increased and integrated telemedicine consultations in their services [39,40]. This implies that users’ experience of the value of telemedicine consultations is confirmed, but how they are used needs further study. The consultations were experienced as time friendly (less waiting time, less travelling time and less time away from work/school) and environmentally friendly (less travelling). Some users reasoned that telemedicine services were a way to off-load conventional primary care to favour patients with more complex health care needs. Therefore, there are potential savings on human and environmental resources, contributing to healthy and sustainable living.

### Care patterns

The care patterns from our study show that 90% of the users had telemedicine consultations with physicians but no other professions. Of those, 76% of patients had participated in only one telemedicine consultation with a physician. The utilization of other health care contacts before or after the telemedicine consultation were surprisingly low. This could be interpreted as telemedicine consultations meeting the health care needs of the users. The three most common diagnoses found in the register data were infections, dermatological complaints, and the need for prescriptions. This result is in agreement with Ekman et al.’s study with data from an earlier period (2016–2017) [21]. It may be that the health problems or concerns arising in telemedicine consultations might be less severe, and it would be of interest to study this because the Covid-19 pandemic may have changed the users’ reason for using telemedicine consultation. Some of the informants said that more complex health issues were their reason for contacting a physician for telemedicine consultation [7].

Users with complex health issues and with minor health issues can benefit from telemedicine consultations because the issues arising with conventional booking systems and, thus, accessibility to care are minimized [24,28]. Our study showed that accessibility of telemedicine consultations was time saving for users, even though previous studies showed that they were not always time saving for the general practitioner [24,28]. In a study in the Southern Region of Sweden from 2016 to 2018, users of telemedicine consultations also used ordinary primary care more often than other users and acute care facilities as often as other users [15]. Comorbidities were not considered in their analyses, and it is not clear if the telemedicine users had increased morbidity compared with conventional primary care users.

The telemedicine care users in our study rarely used 1177 Health Care Guidance with nurses before telemedicine: the users considered their health care needs to be better met by a telemedicine consultation with a physician. A triage process is integrated within the three telemedicine consultation services in this study, so the patients who are assigned a physician are screened as part of the telemedicine consultation. Previous research showed that telemedicine consultations with physicians decreased physical primary care meetings with nurses [15]. The users’ belief in their own capacity to address the type of care that they needed should be acknowledged, because individuals are responsible for their own health. Thus, the accessibility of telemedicine consultations can be time saving for the user. There are conflicting results from studies on whether these kinds of health care services are timesaving and effective for physicians in primary care [6,24,28]. Our study was about telemedicine consultations with physicians, but during the study, it became evident that there is an increasing trend in telemedicine consultations with other professions, and digital solutions should triage patients to the appropriate professionals, not only physicians.

### Strengths and limitations of the study

The mixed methods design strengthens the credibility of the results [34,41,42]; the qualitative data added valuable knowledge and supported the interpretation of the quantitative data. We used purposive sampling regarding age, gender, urban or rural living across Sweden for the qualitative data collection, however, not for attitudes to telemedicine. This study needs to be complemented by studies on primary care users choosing to not use telemedicine consultations with physicians to explore their reasons for not using the service. This study has not explored ethnicity or socioeconomic factors or health economics in relation to using telemedicine consultations, which is a limitation. However, this study does not deal with whether telemedicine consultations contribute to unnecessary care consumption.

Elderly patients used telemedicine consultations less often than other age groups, and only a few elderly persons were interviewed in this study. The views of elderly persons on telemedicine consultations need to be explored further. Telemedicine services may need to be adapted to better suit the elderly population, as claimed in some Swedish studies [40,43]. Future research should preferably be interdisciplinary [40] and it would be beneficial to include the needs and competences of various user groups in using digital tools and services, as well as limitations and novel opportunities for the use of telemedicine in primary care.

## Conclusions and implications

The users experienced that telemedicine consultations met their current health care needs and provided accessibility. They were satisfied with the service and felt competent to choose when these consultations were appropriate. Telemedicine consultations were perceived to be safe and save resources for individuals, the health care system, and the environment. Few of the telemedicine consultations with a physician led to further physical contacts in primary care or in acute care. Users experienced that telemedicine consultants provided an alternative care service for less severe health problems. Given that users mostly had a positive experience, telemedicine consultations in primary care are a valuable service that could be further expanded to meet the needs of other population groups, such as elderly patients.
